# The Relationship Between Preoperative Patient-Reported Outcomes Measurement Information System (PROMIS) Pain Interference and Pain Intensity Scores and Early Postoperative Pain and Opioid Consumption After Lumbar Fusion

**DOI:** 10.7759/cureus.55335

**Published:** 2024-03-01

**Authors:** Parimal Rana, Jane C Brennan, Andrea H Johnson, Justin J Turcotte, Chad Patton

**Affiliations:** 1 Orthopedic Research, Anne Arundel Medical Center, Annapolis, USA; 2 Orthopedics, Anne Arundel Medical Center, Annapolis, USA; 3 Orthopedic Surgery, Anne Arundel Medical Center, Annapolis, USA

**Keywords:** lumbar fusion surgery, postoperative opioid use, promis scores, promis pain intensity, promis pain interference

## Abstract

Background

The Patient-Reported Outcomes Measurement Information System (PROMIS) pain interference and pain intensity measures quantify separate dimensions of pain from the patient's perspective. This study aimed to assess differences in these outcomes and to evaluate whether baseline PROMIS pain scores could be used as a leading indicator of increased pain and opioid consumption during early recovery after lumbar fusion.

Methods

A retrospective review of 199 consecutive patients undergoing posterolateral fusion (PLF) at a single institution was performed. All patients underwent one to three level lumbar PLF and preoperatively completed the PROMIS pain intensity and PROMIS pain interference measures. Multivariate linear regression was used to assess the relationship between preoperative PROMIS scores and postoperative pain numeric rating scale (NRS) and oral morphine milligram equivalents (OMME) by day after controlling for age, sex, and body mass index (BMI).

Results

In comparison to patients with the lowest preoperative pain intensity scores, those with the highest scores required significantly more OMME on postoperative day (POD) zero and one (both p<0.05) and had higher pain NRS on POD one (p=0.02). Patients with the highest pain interference scores reported higher pain NRS on POD zero (p=0.02) but required similar OMME at all time points. After controlling for age, sex, and BMI, each one-point increase in preoperative PROMIS pain interference scores was associated with increased OMME on POD zero (β=0.29, p=0.04) and POD one (β=0.64, p=0.03).

Conclusions

Patients with high pain intensity reported higher levels of pain and required more opioids during the first 24 hours postoperatively, while those with high pain interference reported higher levels of pain on the day of surgery but utilized similar amounts of opioids. After risk adjustment, increased baseline PROMIS pain interference scores - but not pain intensity - were associated with increased opioid use. These results suggest that both measures should be considered when identifying patients at risk for increased pain and opioid consumption after PLF.

## Introduction

Patients undergoing lumbar fusion surgery frequently struggle with significant levels of postoperative pain, necessitating the use of opioid analgesia for relief. Approximately 20% of patients experience chronic post-surgical pain after spine surgery [1]. There is abundant literature that has shown heightened levels of preoperative pain and prior opioid utilization serve as risk factors for intensified pain levels, decreased physical improvement, and increased opioid utilization postoperatively [2,3]. However, precise aspects of pain that influence these outcomes are not well understood, and better predictability of postoperative pain is needed to optimize care processes.

To address this gap in the literature, this study engages the Patient-Reported Outcomes Measurement Information System (PROMIS) pain interference and pain intensity measures to assess the multiple dimensions of pain from the patient's perspective. The pain interference score examines the degree to which pain disrupts activities of daily living, mental, and social activities. The pain intensity measure gauges the severity of pain with a numerical rating scale [4]. Hence, elevated scores on both evaluations indicate an increased level of pain, which is likely to align with diminished physical capabilities and a lower level of mental well-being in patients preparing for spine surgery.

Previous studies have established that patients undergoing lumbar decompression and fusion procedures who exhibit elevated levels of pain interference prior to surgery are more likely to experience clinically significant improvement in both pain and physical function postoperatively [5]. However, the relationship between preoperative pain interference, intensity, and opioid consumption in the early postoperative period remains uncharted. This study aims to establish disparities in these outcomes between patients characterized by high and low baseline pain intensity and interference levels. Additionally, it seeks to ascertain whether baseline PROMIS pain scores might serve as a predictor for pain and heightened opioid requirements during the initial stages of recovery following lumbar fusion surgery.

## Materials and methods

Study population

A retrospective review of 199 consecutive patients undergoing posterolateral fusion (PLF) with two fellowship-trained spine surgeons at a single institution was performed from January 1, 2021, to December 31, 2022. All patients underwent one to three level lumbar PLF with instrumentation and completed the PROMIS pain intensity and PROMIS pain interference measures preoperatively. The PROMIS surveys were given to patients to complete at each visit. PROMIS pain intensity measures the severity of pain patients are experiencing right now, along with average pain and worst pain experienced. The raw score is converted to a T-score that ranges from 30.8 to 71.8 [4]. PROMIS pain interference measures to what degree pain limits a patient's physical, mental, and social functioning. The raw score is converted to a T-score that ranges from 40.7 to 77.0 [4]. Patients who did not complete PROMIS surveys or who underwent four or more level PLF or cervical or thoracic fusions were excluded. The study was deemed institutional review board exempt by the institution's clinical research committee.

Independent variables

The primary independent variables of interest were the preoperative PROMIS pain intensity and pain interference T-scores. Patient age, body mass index (BMI), sex, race, and American Society of Anesthesiologists (ASA) score were extracted via electronic medical record (EMR).

Outcome measures

The primary endpoints were maximum pain levels reported using the numeric rating scale (NRS) and oral morphine milligram equivalents (OMME) received during postoperative days (POD) zero through five.

Statistical analysis

The 25th, 50th, and 75th percentiles were calculated for preoperative pain intensity and interference. Multivariate linear regression assessed the relationship between preoperative PROMIS scores and postoperative pain NRS and OMME by day after controlling for age, sex, and body mass index (BMI). Average pain scores and OMMEs received were compared between patients with the lowest (≤25th percentile) and highest (≥75th percentile) levels of PROMIS pain interference and pain intensity prior to surgery using independent samples t-tests. All statistical analyses were performed using R Studio (Version 4.2.2, Posit Software, Boston, US). Statistical significance was assessed at p<0.05.

## Results

On average, patients were 66.2 years old and had a BMI of 31.3 kg/m^2^. Sixty-one percent of patients were female, and 56% had an ASA score ≥3. Forty-nine percent of patients underwent one level, 33% underwent two level, and 18% underwent three level PLF. The 25th and 75th percentile cutoffs of preoperative PROMIS pain intensity and pain interference scores were 52.1, 60.5, 62.1, and 73.5, respectively (Table 1).

**Table 1:** Demographics and preoperative measures ASA - American Society of Anesthesiologists; PROMIS - Patient-Reported Outcomes Measurement Information System Pain intensity range: 30.8-71.8; pain interference range: 40.7-77.0

Variables	All patients, n (%), mean±SD (n=199)
Age, years	66.24±11.39
BMI, kg/m^2^	31.31±5.76
Sex	
Female	121 (60.8)
Male	78 (39.2)
Non-white race	33 (16.6)
ASA 3+	112 (56.3)
Number of levels fused	
1	97 (47.7)
2	66 (33.2)
3	36 (18.1)
Preoperative PROMIS pain intensity	56.56±8.05
25^th^ percentile	52.1
50^th^ percentile	57.5
75^th^ percentile	60.5
Preoperative PROMIS pain interference	66.82±7.00
25^th^ percentile	62.1
50^th^ percentile	66.2
75^th^ percentile	73.5

**Figure 1:**
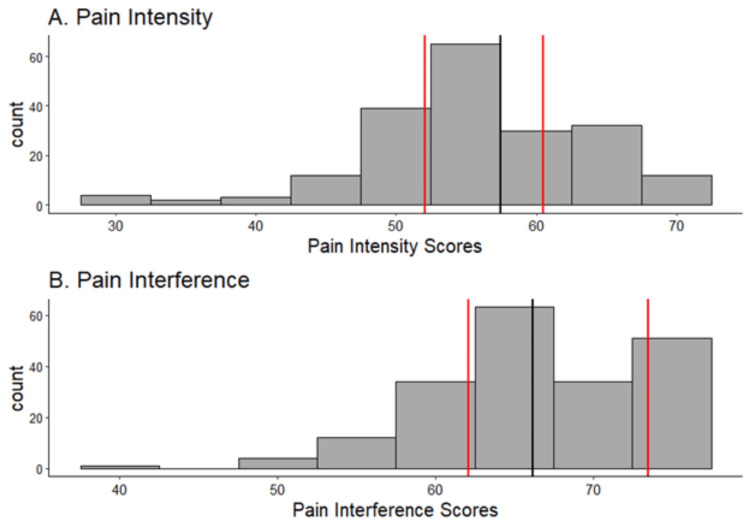
Preoperative pain intensity and interference scores The red lines show the 25th and 75th percentiles and the black line shows the 50th percentile.

Both pain intensity score and pain interference score distributions were skewed to the right (Figure 1).

**Figure 2:**
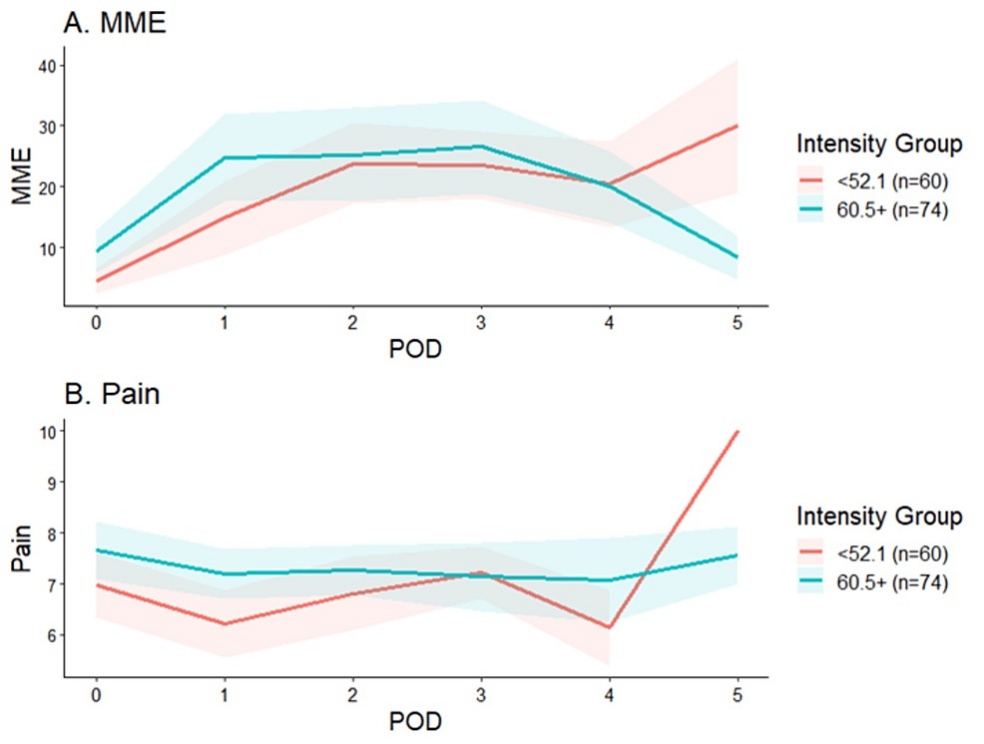
Pain scores and OMME by day by pain intensity The red lines represent the 25^th ^percentile and the green lines represent the 75^th ^percentile. B: Pain on the y-axis refers to the numeric rating scale (NRS) MME - morphine milligram equivalents; POD - postoperative day; OMME - oral morphine milligram equivalents

In comparison to patients with the lowest preoperative pain intensity scores, those with the highest scores required significantly more OMME on POD zero (25th percentile: 4.5 vs. 75th percentile: 9.4 OMME; p=0.02) and one (25th percentile: 14.9 vs. 75th percentile: 24.8 OMME; p=0.04) and had higher pain NRS on POD one (25th percentile: 6.2 vs. 75th percentile: 7.2; p=0.02; Figure 2).

**Figure 3:**
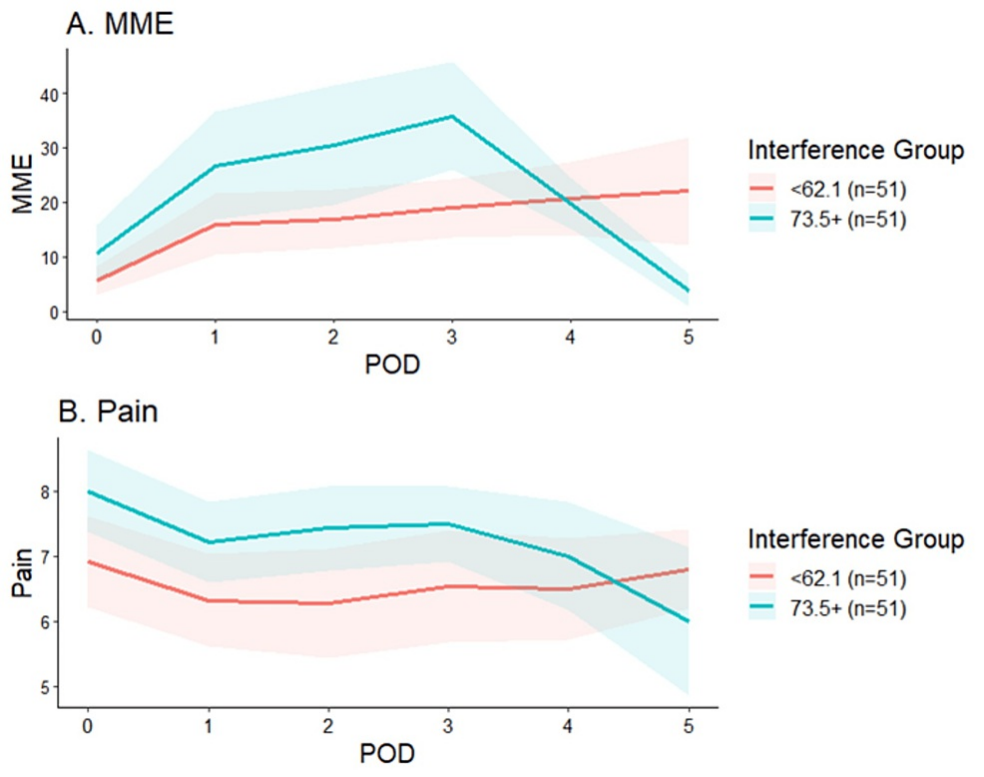
Pain scores and MME by day by pain interference The red lines represent the 25th percentile and the green lines represent the 75th percentile. B: pain on the y-axis refers to the numeric rating scale (NRS) MME - morphine milligram equivalents; POD - postoperative day

Patients with the highest pain interference scores reported higher pain NRS on POD zero (25th percentile: 6.9 vs. 75th percentile: 8.0; p=0.02; Figure 3) but required similar OMME at all time points. 

After controlling for age, sex, and BMI, each one-point increase in preoperative PROMIS pain interference scores was associated with increased OMME on POD zero (β=0.29, 95% confidence interval: 0.02-0.56; p=0.04) and POD one (β=0.64, 95% confidence interval: 0.03-1.26; p=0.03; Table 2).

**Table 2:** Pain interference and intensity as predictors of postoperative pain and MME Data expressed as β (p-value), β - represents the estimated change in the postoperative pain/MME for a one-unit change in pain intensity/interference, controlling for age, sex, BMI; p-value <0.05 is in bold POD - postoperative day; MME - morphine milligram equivalent

Pain intensity/ interference	POD 0		POD 1		POD 2		POD 3		POD 4		POD 5	
	Pain score	MME	Pain score	MME	Pain score	MME	Pain score	MME	Pain score	MME	Pain score	MME
Pain intensity	0.03 (0.18)	0.23 (0.07)	0.02 (0.27)	0.42 (0.13)	0.01 (0.66)	0.03 (0.93)	0.00 (0.93)	0.36 (0.36)	0.07 (0.20)	0.55 (0.15)	0.01 (0.89)	0.44 (0.65)
Pain interference	0.03 (0.25)	0.29 (0.04)	0.02 (0.30)	0.64 (0.03)	0.03 (0.28)	0.54 (0.16)	0.02 (0.59)	0.82 (0.06)	-0.01 (0.87)	0.65 (0.15)	-0.08 (0.38)	0.30 (0.71)

No other statistically significant relationships between preoperative levels of pain interference or intensity and postoperative pain or opioid consumption were observed. 

## Discussion

In the current study, we found that increased baseline PROMIS pain interference, but not pain intensity, scores were associated with increased opioid use on postoperative days zero and one, although not to clinically significant levels. Collectively these findings highlight the multidimensional aspects of pain and the significant variability in how pain is experienced and perceived across individuals undergoing lumbar fusion surgery.

The Veterans Affairs system launched the "Pain as the Fifth Vital Sign" campaign in 1998, focusing a spotlight on patients' perception of pain [6]. Since then, multiple patient-reported scoring systems commonly employed in spine surgery have incorporated an element for evaluating pain have been studied, such as the Visual Analog Scale (VAS), Catastrophizing Pain Scale, Oswestry Disability Index (ODI), SF-36, Roland-Morris Questionnaire, Likert Pain Scale, Odom score, and Nurick scale [7,8]. Hébert et al. showed a worse ODI to be helpful in predicting worse postoperative pain and outcomes [9]. Kleinstück et al. also established that patients' baseline Likert Pain scores predict worse outcomes after decompression surgery [10]. Dunn et al., on the other hand, found that using the Catastrophizing Pain Scale, higher pain scores did not affect postoperative opioid utilization, even though they correlated with higher postoperative pain [11]. Most recently, Nie et al. found patients with greater pain interference preoperatively had a greater probability of achieving minimum clinically important differences in postoperative pain interference, pain, and disability outcomes, when compared to patients with lower preoperative pain interference scores [5]. These studies are similar to our findings, demonstrating how pain scoring can be helpful in outcome predictions and pain management algorithms. However, as demonstrated by the results of the current study, preoperative patient-reported assessments of pain intensity and interference do not alone hold the ability to predict postoperative pain-related outcomes. While these measures appear to hold some prognostic value, they must be considered alongside other clinical and psychosocial factors when formulating strategies for postoperative pain management at the individual level.

Preoperative opioid exposure is another significant factor that may influence postoperative pain and outcomes. Patients exposed to opioids prior to their surgical procedure are more likely to continue relying on these medications in the postoperative recovery phase [12-15]. This association highlights the importance of thorough preoperative assessments, where clinicians must weigh the necessity of continued opioid use against potential alternatives for pain management. Preoperative opioid utilization has also been shown to increase rates of one-year reoperations, emergency department visits, epidural and facet joint injections, and wound complications and hinder patient-reported outcomes [3,11,16]. McCurdy et al. found that preoperative opioid use correlated with worse PROMIS scores two years postoperatively, including pain interference and pain intensity, influencing outcomes and the possibility of increased postoperative opioid use [17]. While these studies demonstrate the potential for chronic opioid users to experience suboptimal postoperative outcomes, others have shown spine surgery to effectively reduce patients' dependence on opioids. In a study of 15,573 patients undergoing lumbar decompression and decompression with fusion, Rezaii et al. found that 50% of patients requiring opioid pain management preoperatively were able to discontinue using opioids after surgery [18]. Given the potential risks and benefits of surgery on opioid consumption, the use of tools such as those presented in the current study is warranted to tailor postoperative pain management protocols in an effort to mitigate the risk of prolonged opioid use.

Several studies have explored critical risk factors of postoperative opioid use among spine patients. Age has been identified as a confounding variable in the assessment of PROMIS scores among spine patients grappling with postoperative pain. Studies have shown that older patients report more pain interference, while no difference in interference or intensity was found between genders [19]. Other studies have found females to report greater pain intensity when dealing with spine pain; however, a strength of the current study is its evaluation of the relationship between preoperative PROMIS scores and pain-related outcomes after controlling for both age and sex [20]. Additionally, specific clinical and lifestyle factors have been shown to impact postoperative opioid use substantially. Notably, patients with pre-existing mental health diagnoses, a history of tobacco usage, a diagnosis of chronic pain, and those prescribed non-narcotic neuromodulatory medications are more likely to require prolonged postoperative opioid therapy [12,21]. Anxiety has been found to be a barrier to spine surgery outcomes, as patients have reported increased anxiety due to a lack of clarity regarding postoperative expectations, which has been linked to poorer outcomes after spine surgery [22]. Interestingly, fear of addiction was found to not correlate with less opioid use, potentially due to fear of disease progression being a greater threat [22]. Recognizing these risk factors is pivotal in tailoring comprehensive pain management strategies while using patient-reported tools, with a focus on minimizing opioid reliance where feasible. These findings collectively emphasize the multidimensional nature of postoperative pain management for spine patients.

PROMIS pain scoring tools, including PROMIS pain interference and intensity, have been reported to correlate with outcomes as compared to legacy measures in various validity studies [23,24]. The clinical validity of these tools has been explored across various specialties, showing scores to be sensitive to changes in pain, making it a useful tool in the realm of pain management [25]. PROMIS pain interference also has a significantly stronger correlation to predicting outcomes when compared to other measurements like the Likert Pain scale and the Oswestry Disability Index [8,26]. Previous studies have also confirmed a strong negative correlation between self-reported physical function and pain interference [27]. These findings infer that PROMIS pain interference scoring may be a suitable predictor for patient function and outcome. Relating back to pain management, patients with higher scores tend to have significantly elevated opioid use, similar to what we saw in our risk-adjusted cohort [28].

The current study is limited by its retrospective design at a single institution and the potential existence of unmeasured confounding variables. The findings of the current study represent patients within the geographical region of a single institution and may not be applicable to the broader population of patients undergoing lumbar fusion surgery. In addition, it is possible that reporting bias affected the study's results if patients who completed the PROMIS surveys differed from those who did not. Further, although we adjusted for some patient characteristics, there are multiple important confounding factors such as the history of opioid use, symptom duration, whether patients underwent a one, two, or three level fusion, and psychosocial and socioeconomic status that were not evaluated in the study. Finally, given the subjective nature of pain and its treatment, it is difficult to determine the extent to which individual perception and reporting influenced both the PROMIS scores reported and postoperative outcomes.

## Conclusions

While the PROMIS pain instruments may prove useful for incorporation into multivariable models aimed at identifying patients at risk for significant postoperative pain and opioid consumption, they do not appear to achieve this goal when used in isolation. While baseline PROMIS pain interference scores were linked to increased opioid use, this association did not reach clinically significant levels. Additionally, it became evident that relying solely on preoperative patient-reported assessments of pain intensity and interference is insufficient for accurately predicting postoperative pain-related outcomes. The study, however, highlights the need for the continued development of more targeted preemptive measures to enhance the outcomes of individuals undergoing lumbar fusion surgery.
